# Serum phosphorus levels and risk of incident dementia

**DOI:** 10.1371/journal.pone.0171377

**Published:** 2017-02-02

**Authors:** Tingting Li, Yan Xie, Benjamin Bowe, Hong Xian, Ziyad Al-Aly

**Affiliations:** 1 Clinical Epidemiology Center, Research and Education Service, VA Saint Louis Health Care System, Saint Louis, Missouri, United States of America; 2 Department of Medicine, Washington University School of Medicine, Saint Louis, Missouri, United States of America; 3 Department of Biostatistics, College for Public Health and Social Justice, Saint Louis University, Saint Louis, Missouri, United States of America; 4 Department of Medicine, Division of Nephrology, VA Saint Louis Health Care System, Saint Louis, Missouri, United States of America; Istituto Di Ricerche Farmacologiche Mario Negri, ITALY

## Abstract

Higher serum phosphorous is associated with cerebral small vessel disease, an important driver of cognitive decline and dementia. Whether serum phosphorous, a potentially modifiable parameter, associates with risk of incident dementia is not known. We aimed to examine the association between serum phosphorous and risk of incident dementia and to determine if the association is modified by age. We used the United States Department of Veterans Affairs national databases to build a longitudinal observational cohort of US veterans without prior history of dementia and with at least one outpatient serum phosphorus between October 2008 and September 2010 and followed them until September 2014. Serum phosphorus was categorized into quintiles: ≤2.9, >2.9 to ≤3.2, >3.2 to ≤3.5, >3.5 to ≤3.9, >3.9 mg/dL. There were 744,235 participants in the overall cohort. Over a median follow-up of 5.07 years (Interquartile range [IQR]: 4.28, 5.63), adjusted Cox models show that compared to quintile 2, the risk of incident dementia was increased in quintile 4 (Hazard Ratio [HR] = 1.05; CI = 1.01–1.10) and quintile 5 (HR = 1.14; CI = 1.09–1.20). In cohort participants ≤60 years old, the risk of incident dementia was increased in quintile 4 (HR = 1.29; CI = 1.12–1.49) and 5 (HR = 1.45; CI = 1.26–1.68). In participants > 60 years old, the risk was not significant in quintile 4, and was attenuated in quintile 5 (HR = 1.10; CI = 1.05–1.16). Formal interaction analyses showed that the association between phosphorous and dementia was more pronounced in those younger than 60, and attenuated in those older than 60 (P for interaction was 0.004 and <0.0001 in quintiles 4 and 5; respectively). We conclude that higher serum phosphorous is associated with increased risk of incident dementia. This association is stronger in younger cohort participants. The identification of serum phosphorous as a risk factor for incident dementia has public health relevance and might inform the design and implementation of risk reduction strategies.

## Introduction

Dementia is a very important public health problem in the general population. It has been associated with increased mortality, and contributes substantially to rising health care costs [1]. Although studies have shown that age-adjusted incidence of dementia is decreasing in the United States, the overall national prevalence of dementia remains substantially high [2, 3]. A better understanding of potentially modifiable risk factors associated with increased risk of dementia might inform strategies of reducing its incidence.

Dementia is an increasingly recognized comorbidity in patients with chronic kidney disease (CKD), a chronic disease state characterized by disturbance in phosphorus homeostasis where serum phosphorus is often increased [1, 4–8]. Recent observations by Murray and collaborators who examined risk factors for cognitive impairment in a cross sectional study of 422 community-dwelling cohort participants with CKD suggest that elevation in serum phosphorus levels (≥4.5 mg/dL) was associated with substantial risk of cognitive impairment [9]. Observations by Wright and collaborators suggest that higher Fibroblast Growth Factor 23 (FGF-23)—a bone-derived hormone that regulates phosphorus homeostasis and that is often elevated in patients with CKD—is associated with increased risk of stroke even in patients with normal kidney function [10]. As elevation of FGF-23 usually precedes that of serum phosphorus, the authors hypothesized that FGF-23 might be elevated in some patients with normal kidney function as a response to increased dietary intake of phosphorus and might confer increased risk of stroke [10]. The same group of investigators also established an association between FGF-23 and white matter hyperintensities and subclinical brain infarction (both seen on magnetic resonance imaging) suggesting a strong association between disordered phosphorus homeostasis and cerebral small vessel disease which is an important driver of cognitive decline and dementia [11, 12]. Observations from the Atherosclerosis Risk in Communities (ARIC) cohort suggest that higher serum phosphorus levels are associated with increased risk of subclinical carotid atherosclerosis and stroke in a general population cohort [13, 14].

Whether serum phosphorus levels are associated with risk of incident dementia has not been examined in large cohort studies. Given that serum phosphorus is a risk factor for cognitive decline in CKD and that higher FGF-23—often elevated in the context of higher serum phosphorus—represents a risk factor for cerebral small vessel disease, we hypothesized that mild elevation in serum phosphorus—even when serum phosphorus levels remain within the reference range—may be associated with increased risk of incident dementia. Because risk of dementia increases exponentially with advancing age, we also postulated that if serum phosphorus is an independent risk factor for dementia, then the risk of incident dementia would be modified by age and more pronounced in younger individuals. To test our hypothesis, we used Department of Veterans Affairs National databases to build a cohort of patients without dementia at baseline and endeavored to examine the association between serum phosphorus and risk of incident dementia and formally evaluate potential interaction due to age.

## Materials and methods

### Patients

**Fig 1:**
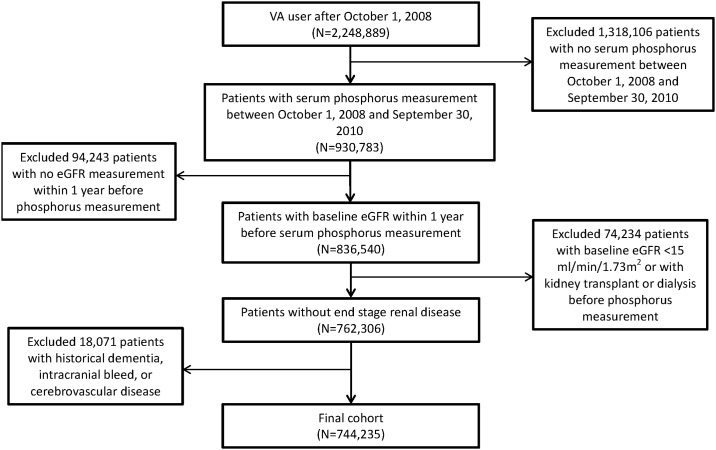
Flow chart for cohort selection.

Using administrative data from the United States Department of Veterans Affairs (VA), we identified users of the VA Healthcare System with at least one outpatient serum phosphorus measurement between October 1, 2008 and September 30, 2010 (n = 930,783), where the date the first phosphorus measurement was designated as the time of cohort entry (time zero (T0)). Cohort participants were also required to have at least one outpatient estimated glomerular filtration rate (eGFR) measurement within 1 year prior to T0 as baseline eGFR (n = 836,540). Participants were included in the cohort only if their baseline eGFR was ≥ 15 mL/min/1.73 m^2^ and did not receive kidney transplant or dialysis before T0 (n = 762,306). Participants were excluded if they were diagnosed with dementia, intracranial bleed, or cerebral vascular accident before T0, yielding an analytic cohort of 744,235. Flow chart for the cohort selection is depicted in Fig 1. The study (#1163689) was approved by the Institutional Review Board (IRB) of the VA Saint Louis Health Care System, Saint Louis, MO. A waiver of informed consent was approved by the IRB.

### Data sources

We used the Department of Veterans Affairs databases including inpatient and outpatient medical SAS datasets (that include utilization data related to all inpatient and outpatient encounters within the VA system) to ascertain detailed patient demographic characteristics and comorbidity information based on Current Procedural Terminology (CPT) codes, ICD-9-CM diagnostic and procedure codes associated with inpatient and outpatient encounters [15, 16]. The VA Managerial Cost Accounting Laboratory Results file (a comprehensive database that includes VA-wide results for selected laboratory tests obtained in the clinical setting) provided information on outpatient serum phosphorus and serum creatinine measurements and other laboratory data. The VA Vital Status and Beneficiary Identification Records Locator Subsystem (BIRLS) files provided demographic characteristics and death follow-up through September 30, 2014. The United States Renal Database System (USRDS) data provided information on date of first kidney transplant and dialysis services. The VHA's Corporate Data Warehouse Vital Signs domains provided data on height and weight to calculate body mass index. County level socioeconomic data in year 2009 were obtained from the 2014–2015 Area Health Resources Files (AHRF) system [17].

### Primary outcomes

The primary outcome was incident dementia. We used validated case definition for dementia based on inpatient and outpatient ICD-9-CM diagnostic codes [18, 19]. These ICD-9 codes, which were validated in population-based studies using administrative health data, had a sensitivity of 69%, specificity of 85%, positive predictive value of 81%, and negative predictive value of 74% for dementia [18, 19]. Outcomes were ascertained from time of cohort entry (T0) until September 30, 2014.

### Primary predictor

The primary predictor was outpatient serum phosphorus. First phosphorus measurement between October 1, 2008 and September 30, 2010 was considered the baseline phosphorus. Patients were grouped into 5 groups based on phosphorus quintiles. Patients with phosphorus values at quintile cut-points were grouped into the lower quintile (hence not all quintiles are exactly 20% of the overall cohort).

### Covariates

Baseline covariates were captured during the 5-year period preceding cohort entry (T0) and included baseline eGFR, number of eGFR measurements, number of hospitalizations, age, race, gender, diabetes mellitus, cardiovascular disease, peripheral artery disease, hypertension, atrial fibrillation, depression and liver cirrhosis[20, 21]. Race/ethnicity was categorized as white, black, or other (Latino, Asian, Native American, or other racial/ethnic minority groups). Comorbidities were assigned on the basis of relevant ICD-9-CM diagnostic and procedures codes and CPT codes in the VA Medical SAS datasets as described previously [22–27]. eGFR was calculated using the abbreviated four-variable Chronic Kidney Disease Epidemiology Collaboration equation based on age, sex, race, and outpatient serum creatinine [28].

### Statistical analysis

Frequencies and proportions for categorical variables, means and standard errors or medians and interquartile ranges for continuous variables were computed. Adjusted incident rates were calculated based on Poisson regression given the same age and gender distributions as the overall cohort. Confidence intervals for incident rates were estimated from normal distributions. Restricted cubic regression spline analyses were undertaken to characterize the shape of the relationship between serum phosphorus and risk of dementia. To account for potential highly influential values, those with serum phosphorus values in the lowest 0.5% and highest 0.5% were excluded from the spline analyses. Hazard ratios of dementia between different phosphorus quintiles were evaluated from multivariate Cox survival models and quintile 2, which has the lowest adjusted incident dementia, was selected as the reference group. Cox model was adjusted for age, race, gender, diabetes mellitus, cardiovascular disease, peripheral artery disease, hypertension, atrial fibrillation, depression, liver cirrhosis, baseline eGFR, number of eGFR measurements and number of hospitalizations. Interaction between age and phosphorus quintiles was added to detect how age modifies the relationship between phosphorus and dementia. To evaluate the relationship between phosphorus and dementia in different sub-populations, we built models within patients with age ≤ 60 and > 60. In addition, all analyses were additionally run with serum phosphorus deciles as the primary predictor. Patients who died without dementia during follow up were considered to have experienced competing risk to dementia and were censored as no dementia at the time of death. We further examined the association between serum phosphorus and risk of dementia subtypes including risks of Alzheimer’s disease, vascular dementia, Lewy body dementia and frontotemporal dementia. Dementia subtypes were defined based on ICD9 codes. In survival analyses, a 95% confidence interval (CI) of a hazard ratio (HR) that does not include unity was considered statistically significant. In all analyses a P-value of 0.05 or less was considered statistically significant. Analyses were performed using SAS Enterprise Guide version 7.1 (SAS Institute, Cary, NC) and R studio version 0.99.891.

### Sensitivity analyses

To evaluate the robustness of the study results, we examined the associations in multiple sensitivity analyses as follows: a. to increase the specificity of the dementia diagnosis, we required the occurrence of at least 2 ICD-9 codes for dementia in 2 separate clinical encounters (where sensitivity and specificity have been shown to be 63% and 88%, respectively, and positive predictive value and negative predictive value were 83% and 72% respectively) [18, 19]; b. to eliminate the effect of Parkinson’s disease on the outcome of dementia, we excluded 2091 patients with Parkinson’s disease who were included in the primary analysis (N = 742,144); c. We made different assumptions for relationships between the effect of phosphorus on dementia and its competing risk death: 1) In order to account for the possibility that the observed relationship between phosphorus and dementia was due to the effect of phosphorus on death, we examined the associations in Fine and Gray models where cohort participants who experienced the competing risk were considered to be at risk for dementia after dead,[29] 2) Assuming death informs the occurrence of dementia and patients who experienced death could also have had dementia, we created a composite outcome of dementia or death, and 3) To evaluate the relationship only in participants who did not experience the competing risk, we excluded participants who died before the occurrence of dementia from the cohort yielding an N = 597,072; d. We additionally built models where we used average serum phosphorus in one year as baseline phosphorus to account for intra-individual variability in serum phosphorus; e. where data was available, and to account for potential contribution of neighborhood factors to the risk of incident dementia, we considered county level characteristics including percentage with high school diploma (N = 742,741) and percentage below poverty level (N = 737,536) as covariates in the models; f. we examined the associations in subcohorts where data on body mass index (N = 727,435), serum calcium (N = 729,274), lipid parameters including HDL-C, LDL-C, and triglycerides (N = 692,777) were available; g. we included all of the above covariates from models e and f in the models (N = 675,484).

## Results

There were 744,235 participants in the overall cohort. The demographic and health characteristics of the overall cohort and according to quintiles of serum phosphorus levels are described in Table 1.

**Table 1:** Baseline demographic and health characteristics of cohort participants.

		Overall	Quintile 1 Phosphorus ≤2.9 mg/dL	Quintile 2 Phosphorus >2.9 and ≤3.2 mg/dL	Quintile 3 Phosphorus >3.2 and ≤3.5 mg/dL	Quintile 4 Phosphorus >3.5 and ≤3.9 mg/dL	Quintile 5 Phosphorus >3.9 mg/dL
N (%)		744235	174430 (23.44)	142894 (19.20)	152113 (20.44)	154856 (20.81)	119942 (16.12)
Age (SD)		62.68 (14.52)	63.37 (13.89)	63.78 (14.19)	63.22 (14.55)	62.17 (14.85)	60.35 (15.02)
Mean Serum Phosphorus in mg/dL (SD)		3.38 (0.62)	2.61 (0.30)	3.11 (0.08)	3.40 (0.08)	3.73 (0.11)	4.35 (0.43)
Baseline eGFR in mL/min /1.73m^2^ (SD)		75.91 (24.10)	77.05 (21.98)	76.37 (22.55)	76.38 (23.32)	76.14 (24.69)	72.81 (28.43)
Gender	Male (%)	693418 (93.17)	166638 (95.53)	135070 (94.52)	141980 (93.34)	141849 (91.60)	107881 (89.94)
	Female (%)	50817 (6.83)	7792 (4.47)	7824 (5.48)	10133 (6.66)	13007 (8.40)	12061 (10.06)
Race	White (%)	563180 (75.67)	135584 (77.73)	110198 (77.12)	115644 (76.03)	115104 (74.33)	86650 (72.24)
	Black (%)	140019 (18.81)	29450 (21.03)	24799 (17.35)	27916 (18.35)	31157 (20.12)	26697 (22.26)
	Other (%)	41036 (5.51)	9396 (5.39)	7897 (5.53)	8553 (5.62)	8595 (5.55)	6595 (5.50)
Cardiovascular disease (%)		322651 (43.35)	72487 (41.56)	60665 (42.45)	65710 (43.20)	68367 (44.15)	55422 (46.21)
Diabetes mellitus (%)		332301 (44.65)	71529 (41.01)	60828 (42.57)	67208 (44.18)	71761 (46.34)	60975 (50.84)
Hypertension (%)		613897 (82.49)	142014 (81.42)	117201 (82.02)	125569 (82.55)	128264 (82.83)	100849 (84.08)
Peripheral artery disease (%)		132786 (17.84)	28968 (16.61)	24502 (17.15)	27155 (17.85)	28760 (18.57)	23401 (19.51)
Atrial fibrillation (%)		87102 (11.70)	21081 (12.09)	16754 (11.72)	17337 (11.40)	17709 (11.44)	14221 (11.86)
Depression (%)		108628 (14.60)	25602 (14.68)	19822 (13.87)	21224 (13.95)	22606 (14.06)	19374 (16.15)
Liver cirrhosis (%)		36931 (4.96)	10331 (5.92)	6553 (4.59)	6603 (4.34)	6946 (4.49)	6498 (5.42)
Number of eGFR measurement (SD)		9.30 (8.82)	9.45 (8.79)	9.02 (8.38)	8.99 (8.55)	9.18 (8.72)	9.95 (9.76)
Number of hospitalization (SD)		0.64 (1.72)	0.64 (1.70)	0.57 (1.60)	0.57 (1.63)	0.63 (1.69)	0.84 (2.03)
Age and gender adjusted incident death (CI)^a^		50.46 (50.22, 50.70)	45.11 (44.65, 45.57)	44.06 (43.55, 44.57)	46.51 (46.00, 47.02)	52.81 (52.27, 53.35)	72.69 (71.96, 73.42)
Age and gender adjusted incident dementia (CI)^a^		6.71 (6.62, 6.80)	7.00 (6.82, 7.18)	6.26 (6.07, 6.45)	6.40 (6.21, 6.59)	6.62 (6.43, 6.81)	7.33 (7.10, 7.56)
Years of follow up (IQR)^b^		5.07 (4.28, 5.63)	5.09 (4.31, 5.63)	5.10 (4.32, 5.63)	5.09 (4.31, 5.63)	5.05 (4.27, 5.63)	4.97 (4.15, 5.60)
Variables for sensitivity analyses							
Parkinson’s disease (%)		2091 (0.28)	551 (0.32)	431 (0.30)	451 (0.30)	406 (0.26)	252 (0.21)
Average Phosphorus ^c^	Number of Phosphorus measure (SD)	1.98 (2.19)	1.70 (1.85)	1.96 (2.14)	2.06 (2.29)	2.08 (2.21)	2.11 (2.37)
	Phosphorus in mg/dL (SD)	3.39 (0.58)	2.63 (0.27)	3.09 (0.09)	3.38 (0.09)	3.68 (0.08)	4.20 (0.39)
	Number of patients (%)^d^	727435	170750 (23.47)	139710 (19.21)	148505 (20.41)	151184 (20.78)	117286 (16.12)
Body Mass Index	Underweight (%)	14699 (2.02)	3206 (1.88)	2563 (1.83)	2758 (1.86)	3103 (2.05)	3069 (2.62)
	Normal (%)	155036 (21.31)	34237 (20.05)	28552 (20.44)	31484 (21.20)	33516 (22.17)	27247 (23.23)
	Overweight (%)	261899 (36.00)	63588 (37.24)	51936 (37.17)	54200 (36.50)	53382 (35.31)	38793 (33.08)
	Obesity (%)	295801 (40.66)	69719 (40.83)	56659 (40.55)	60063 (40.45)	61183 (40.47)	48177 (41.08)
Serum Calcium	Number of patients (%)^d^	729274	171060 (23.46)	140029 (19.20)	149001 (20.43)	151680 (20.80)	117504 (16.11)
	Calcium in mg/dL (SD)	9.37 (0.62)	9.33 (0.70)	9.35 (0.78)	9.38 (0.49)	9.41 (0.50)	9.41 (0.57)
Lipid panel	Number of patients (%)^d^	692777	162628 (23.47)	134016 (19.34)	142255 (20.53)	143900 (20.77)	109978 (15.87)
	HDL-C in mg/dL (SD)	43.72 (15.44)	43.12 (14.95)	43.61 (15.02)	43.81 (15.29)	44.11 (15.65)	44.09 (16.52)
	LDL-C in mg/dL (SD)	101.66 (37.06)	101.93 (36.63)	101.90 (36.64)	101.96 (36.57)	101.53 (37.34)	100.73 (38.41)
	Triglycerides in mg/dL (SD)	150.59 (132.09)	151.05 (136.47)	147.05 (125.34)	146.78 (123.22)	149.54 (126.35)	160.50 (150.18)

Abbreviations: eGFR, estimated glomerular filtration rate; HDL-C, High-density lipoprotein cholesterol; LDL-C, Low-density lipoprotein cholesterol; SD, Standard deviation; IQR, Interquartile range; CI, Confidence interval.

^a.^Incidence per 1000 person-years

^b.^Calculated from time zero until death occurrence or September 30, 2014

^c.^Based on phosphorus measurement within one year after first measurement. Quintile cut-points for Average Phosphorus were 2.9, 3.2, 3.5, and 3.8 mg/dL.

^d.^Patients with related data available

**Fig 2:**
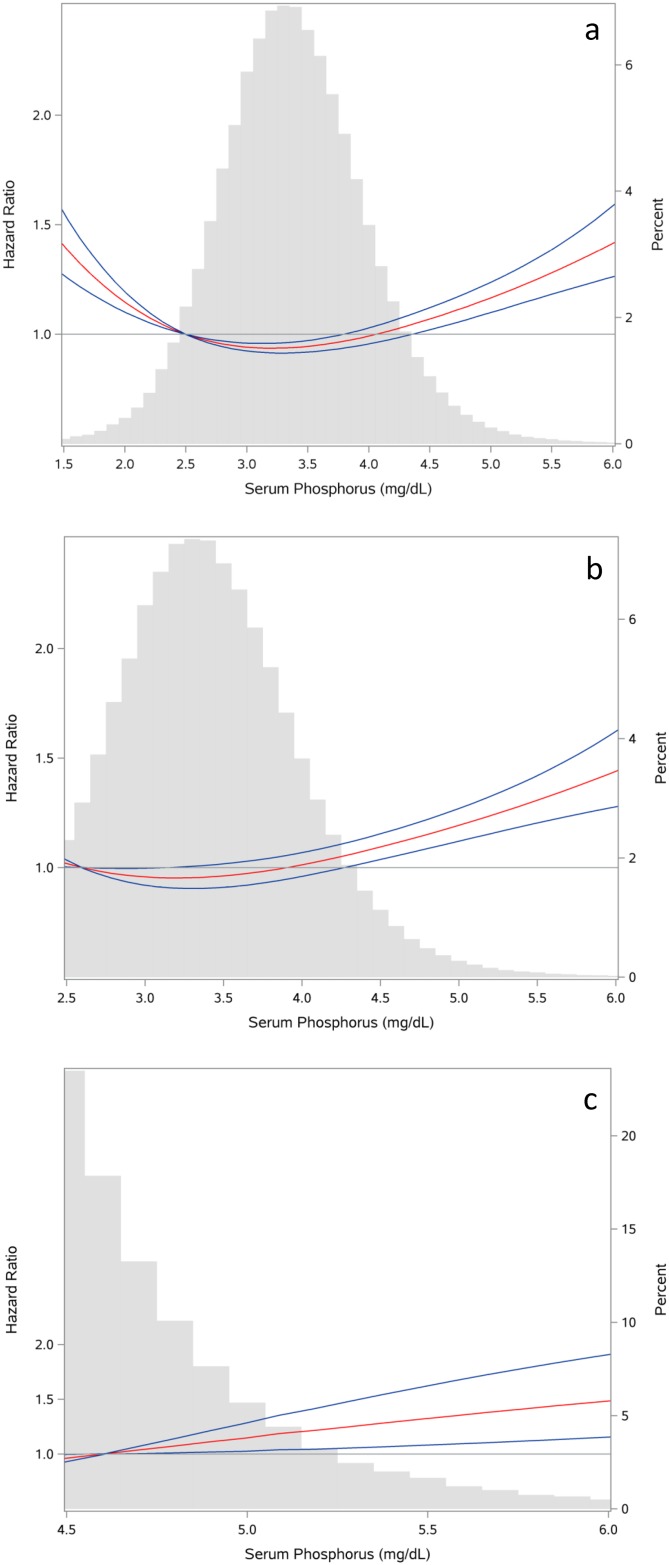
Spline analyses for the relationship between serum phosphorus and risk of incident dementia with serum phosphorus probability distribution histogram represented in gray bars in the background. Fig 2a: Overall cohort. Fig 2b: Analysis was restricted to cohort participants with serum phosphorus level >2.5 mg/dl. Fig 2c: Analysis was restricted to cohort participants with serum phosphorus level >4.5 mg/dl.

Spline analyses depicting the relationship between serum phosphorus and risk of incident dementia are shown in Fig 2. In analyses considering the entire range of serum phosphorus in the overall cohort, there was a U-shaped relationship between phosphorus and risk of incident dementia (Fig 2a). The relationship was not linear in analyses restricted to those with serum phosphorus levels >2.5mg/dl (the lowest range of normal) (Fig 2b). The relationship was linear in those with serum phosphorus levels >4.5 mg/dl (Fig 2c). Given the U-shaped relationship between phosphorus and risk of incident dementia in the overall cohort, we grouped cohort participant into quintiles, and quintile 2 (where the risk was lowest) was designated as the reference category.

### Association between phosphorus and risk of incident dementia

**Fig 3:**
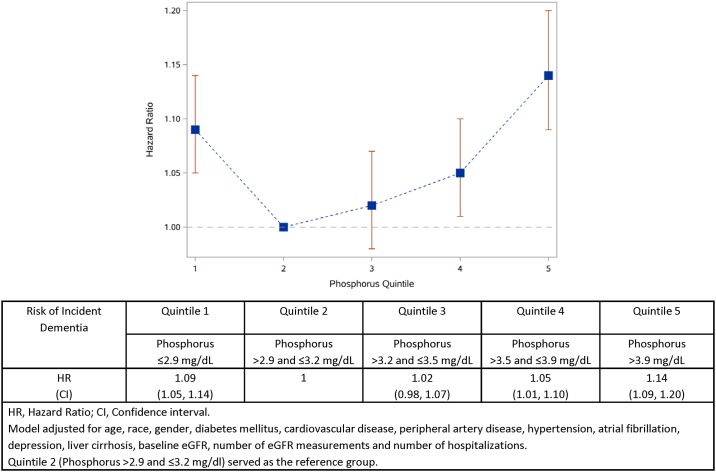
Relationship between serum phosphorus (in quintiles) and risk of incident dementia.

Over a median duration of follow up of 5.07 years (IQR: 4.28, 5.63), the age and gender adjusted rate of incident dementia was 6.26 (6.07–6.45), 6.62 (6.43–6.81), and 7.33 (7.10–7.56) per 1000 person-years in quintile 2 (serum phosphorus > 2.9 and ≤ 3.2 mg/dL), quintile 4 (serum phosphorus > 3.5 and ≤ 3.9 mg/dL), and quintile 5 (serum phosphorus > 3.9 mg/dL), respectively. In analyses adjusted for age, race, gender, diabetes mellitus, cardiovascular disease, peripheral artery disease, hypertension, atrial fibrillation, depression, liver cirrhosis, baseline eGFR, number of eGFR measurements and number of hospitalizations, we evaluated the risk of dementia according to serum phosphorus categorized in quintiles. Compared to quintile 2, the risk of incident dementia was increased in quintile 4 (HR = 1.05; CI = 1.01–1.10) and quintile 5 (HR = 1.14; CI = 1.09–1.20) (Fig 3). We also considered an alternative categorization of serum phosphorus into deciles. Compared to decile 3 (serum phosphorus > 2.9 and ≤ 3.1 mg/dL), the risk of incident dementia was increased in decile 9 (serum phosphorus > 3.9 and ≤ 4.1 mg/dL) [HR = 1.11; CI = 1.04–1.19] and decile 10 (serum phosphorus > 4.1 mg/dL) [HR = 1.17; CI = 1.10–1.24].

### Association of phosphorus and risk of incident dementia is modified by age

**Fig 4:**
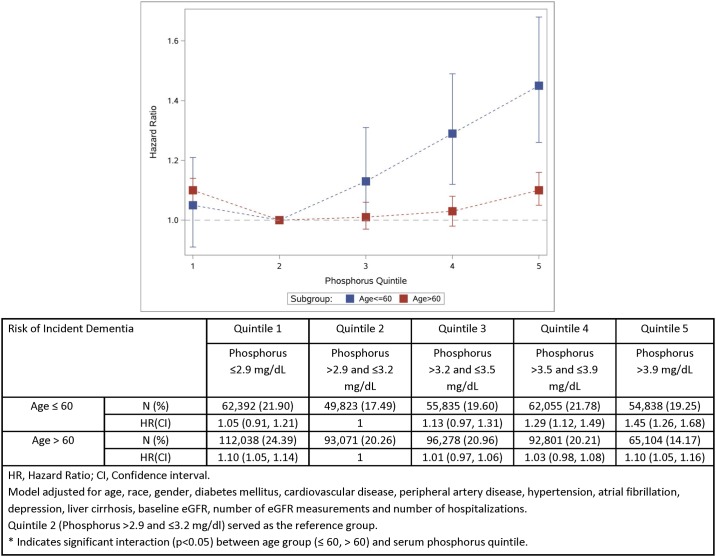
Relationship between serum phosphorus (in quintiles) and risk of incident dementia by age group (≤60 and >60). * Indicates significant interaction (p<0.05) between age group and serum phosphorus in quintiles 4, and 5.

We examined the association between serum phosphorus in quintiles and risk of incident dementia in cohort participants younger and older than 60 years of age. Compared to quintile 2, the risk of incident dementia for participants ≤ 60 years old was increased in quintile 4 (HR = 1.29; CI = 1.12–1.49) and quintile 5 (HR = 1.45; CI = 1.26–1.68) (Fig 4). Risk of incident dementia for participants > 60 years old was not significant in quintile 4 (HR = 1.03; CI = 0.98–1.08) and was attenuated but remained significant in quintile 5 (HR = 1.10; CI = 1.05–1.16) (Fig 4). Formal interaction analyses were undertaken and demonstrated significant effect modification in that age less than or equal to 60 strengthens the association between high level serum phosphorus and risk of incident dementia (P for interaction was 0.004 and <0.0001 in quintiles 4 and 5, respectively). We alternatively categorized age into ≤60, 60–70, and >70, and the results were consistent in that the risk difference between normal and high phosphorus quintiles was more robust in the youngest group, and was attenuated with increasing age (S1 Table). Examination of interaction where age was treated as a continuous variable yielded consistent results (P value for interaction between age and phosphorus quintiles in quintile 4 and 5 were 0.003 and <0.0001, respectively).

### Association between serum phosphorus and dementia subtypes

**Fig 5:**
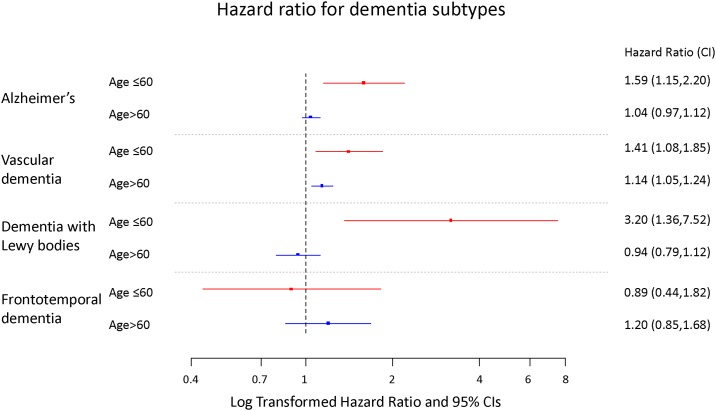
Relationship between elevated serum phosphorus (quintile 5) and risk of dementia subtype by age group (≤60 and >60).

To determine if higher serum phosphorus is associated with a specific subtype of dementia, we undertook analyses to examine the association of serum phosphorus levels and risk of incident Alzheimer’s disease, risk of vascular dementia, risk of Lewy body dementia, and risk of frontotemporal dementia (Table 2) (Fig 5). The results suggest that higher serum phosphorus levels were associated with increased risk of Alzheimer’s disease, vascular dementia, and Lewy body dementia in those younger than 60 years of age. These associations were attenuated and became non-significant in those older than 60 years of age with notable exception of vascular dementia where the association was attenuated but remained statistically significant (Table 2). There was no association between serum phosphorus and risk of frontotemporal dementia in the overall cohort and in analyses stratified by age.

**Table 2:** Association between serum phosphorus and dementia subtypes.

Dementia subtypes			Quintile 1 Phosphorus ≤ 2.9mg/dL	Quintile 2 Phosphorus > 2.9 and ≤ 3.2 mg/dL	Quintile 3 Phosphorus > 3.2 and ≤ 3.5 mg/dL	Quintile 4 Phosphorus > 3.5 and ≤ 3.9 mg/dL	Quintile 5 Phosphorus> 3.9 mg/dL
Alzheimer’s	Overall	HR (CI)	1.07 (1.01, 1.13)	1	1.01 (0.95, 1.07)	1.03 (0.97, 1.10)	1.06 (0.98, 1.13)
	Age ≤ 60	HR (CI)	1.07 (0.77, 1.50)	1	1.37 (0.98, 1.90)	1.63 (1.19, 2.23)	1.59 (1.15, 2.20)
	Age > 60	HR (CI)	1.07 (1.01, 1.13)	1	1.00 (0.94, 1.06)	1.01 (0.95, 1.08)	1.04 (0.97, 1.12)
Vascular dementia	Overall	HR (CI)	1.09 (1.01, 1.19)	1	1.05 (0.98, 1.13)	1.05 (0.97, 1.13)	1.16 (1.07, 1.26)
	Age ≤ 60	HR (CI)	0.77 (0.57, 1.04)	1	1.15 (0.87, 1.52)	1.17 (0.89, 1.53)	1.41 (1.08, 1.85)
	Age > 60	HR (CI)	1.11 (1.03, 1.19)	1	1.05 (0.97, 1.13)	1.04 (0.96, 1.13)	1.14 (1.05, 1.24)
Dementia with Lewy body	Overall	HR (CI)	1.08 (0.94, 1.24)	1	0.96 (0.83, 1.11)	0.94 (0.81, 1.09)	0.98 (0.83, 1.16)
	Age ≤ 60	HR (CI)	2.18 (0.92, 5.15)	1	2.28 (0.94, 5.49)	3.38 (1.47, 7.77)	3.20 (1.36, 7.52)
	Age > 60	HR (CI)	1.05 (0.92, 1.21)	1	0.94 (0.81, 1.09)	0.89 (0.77, 1.04)	0.94 (0.79, 1.12)
Frontotemporal dementia	Overall	HR (CI)	0.94 (0.72, 1.24)	1	0.98 (0.74, 1.30)	0.92 (0.69, 1.23)	1.12 (0.82, 1.52)
	Age ≤ 60	HR (CI)	0.67 (0.34, 1.35)	1	1.17 (0.62, 2.22)	0.64 (0.30, 1.34)	0.89 (0.44, 1.82)
	Age > 60	HR (CI)	1.00 (0.74, 1.34)	1	0.94 (0.69, 1.29)	1.00 (0.73, 1.36)	1.20 (0.85, 1.68)

Abbreviations: HR, hazard ratio; CI, confidence interval.

Model adjusted for age, race, gender, diabetes mellitus, cardiovascular disease, peripheral artery disease, hypertension, atrial fibrillation, depression, liver cirrhosis, baseline eGFR, number of eGFR measurements and number of hospitalizations.

Quintile 2 (phosphorus >2.9 and ≤3.2 mg/dl) served as the reference group.

### Sensitivity analyses

We evaluated the robustness of the study findings in a number of sensitivity analyses (Table 3) where we: a. required the occurrence of 2 ICD-9 codes for dementia for disease definition; b. excluded patients with Parkinson’s disease from the cohort; c. considered death as a competing risk for dementia outcome where we 1) examined the associations in Fine and Gray models, 2) created a composite outcome of dementia or death, 3) excluded participants who died before the occurrence of dementia from cohort; d. to account for potential intra-individual variability in serum phosphorus, we used average serum phosphorus as a covariate; e. to account for potential contribution of neighborhood factors to the risk of incident dementia, we considered county level factors including percentage with high school diploma and percentage below poverty level as covariates in the models; f. included body mass index, serum calcium, and lipid parameters including HDL-C, LDL-C, and triglycerides as covariates in the models; g. included covariates mentioned in e and f in the models. The results of the sensitivity analyses consistently showed an association between higher serum phosphorus and increased risk of incident dementia and that the association was more pronounced in cohort participants younger than 60 years old (Table 3).

**Table 3:** Sensitivity analyses.

Models			Quintile 1 Phosphorus ≤ 2.9 mg/dL	Quintile 2 Phosphorus > 2.9 and ≤ 3.2 mg/dL	Quintile 3 Phosphorus > 3.2 and ≤ 3.5 mg/dL	Quintile 4 Phosphorus > 3.5 and ≤ 3.9 mg/dL	Quintile 5 Phosphorus> 3.9 mg/dL
Use 2 occurrences of Dementia diagnoses as outcome definition	Overall	HR (CI)	1.09 (1.02, 1.16)	1	1.02 (0.95, 1.09)	1.03 (0.96, 1.10)	1.12 (1.04, 1.21)
	Age ≤ 60	HR (CI)	1.14 (0.84, 1.55)	1	1.34 (0.99, 1.82)	1.51 (1.13, 2.04)	1.74 (1.29, 2.35)
	Age > 60	HR (CI)	1.09 (1.01, 1.16)	1	1.00 (0.93, 1.07)	1.00 (0.93, 1.08)	1.09 (1.00, 1.18)
Excluded cohort participants with Parkinson’s disease (N = 742,144)	Overall	HR (CI)	1.09 (1.05, 1.14)	1	1.02 (0.98, 1.07)	1.06 (1.02, 1.11)	1.17 (1.11, 1.23)
	Age ≤ 60	HR (CI)	1.04 (0.90, 1.20)	1	1.08 (0.93, 1.26)	1.25 (1.08, 1.44)	1.44 (1.25, 1.67)
	Age > 60	HR (CI)	1.10 (1.05, 1.15)	1	1.02 (0.97, 1.06)	1.04 (0.99, 1.09)	1.12 (1.07, 1.19)
Fine & Gray model	Overall	HR (CI)	1.09 (1.05, 1.13)	1	1.02 (0.97, 1.06)	1.03 (0.98, 1.07)	1.05 (1.00, 1.10)
	Age ≤ 60	HR (CI)	1.05 (0.91, 1.22)	1	1.13 (0.97, 1.31)	1.28 (1.11, 1.48)	1.41 (1.22, 1.62)
	Age > 60	HR (CI)	1.09 (1.05, 1.14)	1	1.01 (0.96, 1.05)	1.00 (0.95, 1.05)	1.01 (0.96, 1.06)
Composite outcome of Death and Dementia	Overall	HR (CI)	1.02 (1.00, 1.03)	1	1.05 (1.03, 1.06)	1.16 (1.15, 1.18)	1.48 (1.46, 1.51)
	Age ≤ 60	HR (CI)	1.04 (1.00, 1.08)	1	1.05 (1.01, 1.09)	1.16 (1.12, 1.21)	1.57 (1.51, 1.64)
	Age > 60	HR (CI)	1.02 (1.00, 1.03)	1	1.05 (1.03, 1.06)	1.16 (1.14, 1.18)	1.45 (1.42, 1.47)
Excluded dead before dementia occurrence (N = 597,072)	Overall	HR (CI)	1.08 (1.04, 1.12)	1	1.04 (1.00, 1.08)	1.10 (1.05, 1.15)	1.26 (1.21, 1.32)
	Age≤60	HR (CI)	1.04 (0.90, 1.21)	1	1.13 (0.98, 1.31)	1.30 (1.13, 1.50)	1.51 (1.31, 1.74)
	Age>60	HR (CI)	1.09 (1.04, 1.13)	1	1.03 (0.98, 1.07)	1.08 (1.03, 1.13)	1.22 (1.16, 1.29)
Used average phosphorus value within one year^a^	Overall	HR (CI)	1.05 (1.01, 1.09)	1	1.04 (1.00, 1.09)	1.05 (1.01, 1.10)	1.10 (1.05, 1.15)
	Age ≤ 60	HR (CI)	1.02 (0.87, 1.18)	1	1.20 (1.04, 1.38)	1.39 (1.20, 1.60)	1.50 (1.31, 1.72)
	Age > 60	HR (CI)	1.05 (1.01, 1.10)	1	1.03 (0.99, 1.08)	1.02 (0.97, 1.07)	1.05 (1.00, 1.10)
Additionally control for education at county level (N = 742,741)	Overall	HR (CI)	1.09 (1.05, 1.14)	1	1.02 (0.98, 1.07)	1.05 (1.01, 1.10)	1.15 (1.09, 1.20)
	Age ≤ 60	HR (CI)	1.05 (0.91, 1.21)	1	1.13 (0.97, 1.31)	1.29 (1.12, 1.49)	1.45 (1.26, 1.68)
	Age > 60	HR (CI)	1.10 (1.05, 1.15)	1	1.01 (0.97, 1.06)	1.03 (0.98, 1.08)	1.10 (1.05, 1.16)
Additionally control for poverty at county level (N = 737,536)	Overall	HR (CI)	1.09 (1.05, 1.14)	1	1.02 (0.98, 1.07)	1.06 (1.01, 1.11)	1.15 (1.10, 1.21)
	Age ≤ 60	HR (CI)	1.04 (0.90, 1.20)	1	1.14 (0.98, 1.32)	1.30 (1.13, 1.50)	1.45 (1.26, 1.68)
	Age > 60	HR (CI)	1.10 (1.05, 1.15)	1	1.01 (0.97, 1.06)	1.03 (0.99, 1.08)	1.11 (1.05, 1.16)
Additionally control for BMI (N = 727,435)	Overall	HR (CI)	1.09 (1.04, 1.13)	1	1.02 (0.97, 1.06)	1.03 (0.99, 1.08)	1.11 (1.06, 1.16)
	Age ≤ 60	HR (CI)	1.05 (0.91, 1.21)	1	1.11 (0.96, 1.29)	1.25 (1.09, 1.44)	1.35 (1.17, 1.56)
	Age > 60	HR (CI)	1.09 (1.05, 1.14)	1	1.01 (0.96, 1.05)	1.01 (0.96, 1.06)	1.07 (1.02, 1.13)
Additionally control for Calcium level (N = 729,274)	Overall	HR (CI)	1.09 (1.04, 1.13)	1	1.02 (0.98, 1.07)	1.06 (1.01, 1.10)	1.15 (1.09, 1.20)
	Age≤60	HR (CI)	1.04 (0.90, 1.20)	1	1.14 (0.98, 1.33)	1.32 (1.14, 1.52)	1.48 (1.29, 1.71)
	Age>60	HR (CI)	1.10 (1.05, 1.14)	1	1.01 (0.97, 1.06)	1.03 (0.98, 1.08)	1.10 (1.05, 1.16)
Additionally control for HDL-C, LDL-C and Triglyceride (N = 692,777)	Overall	HR (CI)	1.10 (1.06, 1.15)	1	1.03 (0.98, 1.08)	1.06 (1.01, 1.10)	1.13 (1.08, 1.19)
	Age≤60	HR (CI)	1.11 (0.96, 1.30)	1	1.15 (0.99, 1.35)	1.29 (1.11, 1.50)	1.42 (1.22, 1.66)
	Age>60	HR (CI)	1.10 (1.06, 1.15)	1	1.02 (0.97, 1.07)	1.03 (0.99, 1.08)	1.10 (1.04, 1.15)
Additionally control for BMI, education and poverty at county level, Calcium, HDL-C, LDL-C and Triglyceride (N = 675,484)	Overall	HR (CI)	1.10 (1.05, 1.15)	1	1.03 (0.98, 1.08)	1.05 (1.00, 1.09)	1.11 (1.06, 1.17)
	Age≤60	HR (CI)	1.08 (0.93, 1.26)	1	1.15 (0.98, 1.34)	1.26 (1.09, 1.47)	1.34 (1.15, 1.57)
	Age>60	HR (CI)	1.10 (1.05, 1.15)	1	1.02 (0.97, 1.07)	1.02 (0.98, 1.07)	1.08 (1.02, 1.14)

Abbreviations: BMI, body mass index; HDL-C, high density lipoprotein cholesterol; LDL-C, low density lipoprotein cholesterol; HR, hazard ratio; CI, confidence interval.

Model adjusted for age, race, gender, diabetes mellitus, cardiovascular disease, peripheral artery disease, hypertension, atrial fibrillation, depression, liver cirrhosis, baseline eGFR, number of eGFR measurements and number of hospitalizations.

Quintile 2 (phosphorus >2.9 and ≤3.2 mg/dl) served as the reference group.

^a.^ Quintile cut-points for Average Phosphorus were 2.9, 3.2, 3.5, and 3.8 mg/dL.

## Discussion

In this large national cohort of 744,235 veterans, we found that higher serum phosphorus levels were independently associated with increased risk of incident dementia; this association was significantly modified by age and was more pronounced in patients less than 60 years old.

This report suggests a novel and potentially modifiable risk factor for incident dementia in a large cohort of United States veterans. Our results demonstrating that phosphorus is a risk factor for dementia provide important therapeutic potential and public health relevance. The American diet is rich in phosphorus [30]. While the recommended dietary allowance for phosphorus is 700 mg per day, the actual daily consumption by the average American far exceeds this amount [31]. Inorganic phosphate is heavily used in processed foods, is often not listed on food labels, and has high bioavailability. It was reported that 44% of popular grocery items contained phosphorus additives [32] and this hidden source of phosphorus could contribute as much as 1000 mg to an individual’s daily phosphorus consumption [33]. Studies have shown that greater dietary intake of phosphorus results in increased serum phosphorus and FGF-23 levels and lower dietary intake of phosphorus or consumption of diet where bioavailability of phosphorus is low (vegetarian diet) lowers serum phosphorus and FGF-23 [17, 34–36]. Whether strategies aimed at reducing phosphorus intake and resultant attenuation of serum phosphorus levels leads to reduction of dementia risk merits investigation.

Dementia is often considered a disease of old age and risk increases significantly as age increases. That the relationship between serum phosphorus and risk of incident dementia is stronger in younger cohort participants (and the corollary observation that risk is attenuated with increasing age) lends validity to our findings and suggests that the mechanism(s) underpinning the association of serum phosphorus and dementia may be different from those of age-related dementia. An alternative explanation is that risk of dementia substantially increases with age and in older adults the contribution of age to risk estimates dwarfs other important but less strong risk factors. In our analyses, we found that higher serum phosphorus was associated with increased risk of Alzheimer’s disease, vascular dementia, and Lewy body dementia. Although plausible, our results do not endorse the hypothesis that serum phosphorus may be differentially associated with specific subtype of dementia.

In this report, there was a slightly increased risk of incident dementia in quintile 1 of the overall cohort (those with serum phosphorus ≤ 2.9 mg/dL) [HR = 1.09; CI = 1.05–1.14]. In stratified analyses by age (> 60 and ≤ 60 years old), the risk was not increased among cohort participants younger than 60 years old (HR = 1.05; CI = 0.91–1.12), and slightly increased in those older than 60 (1.10; CI = 1.05–1.14). That the association was only significant in those older than 60 years old may be explained by residual confounding—the notion that older adults with low serum phosphorus may also have other illnesses (or confounders), both unmeasured and unknown, that might explain the slight increase in risk in dementia. The prevalence of malnutrition is very high in the elderly, even in the developed countries [37, 38]. Previous studies have shown that poor nutritional status was significantly associated with faster cognitive decline [39, 40]. Low phosphorus levels can be a consequence of malnutrition and may explain the observed association between low phosphorus and dementia in those > 60 years old.

To our knowledge, this is the first population-based study evaluating the association between serum phosphorus levels and the risk of incident dementia. The pathophysiologic mechanisms underlying the observed association are not clear. Higher serum phosphorus levels are associated with increased risks of cardiovascular events and mortality in patients with normal kidney function; similar associations have been described in persons who consume a high phosphorus diet [31, 41–44]. Elevated phosphorus levels have also been shown to be associated with increased carotid intima-media thickness independent of traditional atherosclerotic risk factors [13]. In two separate community-based cohorts, subclinical atherosclerotic calcification was found to be associated with impaired cognitive function and atherosclerotic disease accounted for significant cognitive impairment [45, 46]. Other reports have suggested that atherosclerotic carotid disease is not only a major contributor to “vascular” dementia, it may also play an important role in the pathogenesis of Alzheimer’s disease and other subtypes of cognitive impairment [5, 47–49]. Increased FGF-23 which occurs in the setting of excess phosphorus intake may be a mediator or contributor to the observed adverse outcomes [10, 50]. Higher serum phosphorus concentration has been shown *in vitro* and *in vivo* to cause endothelial dysfunction via increased production of reactive oxygen species, decreased nitric oxide release/availability, increased oxidative stress and inflammatory response, leading to dysregulation of vascular tone [51, 52]. Hyperphosphatemia can also induce the release of endothelial membrane-derived microparticles (MPs) which are markers of vascular dysfunction [53, 54]. These MPs are known to have procoagulant effects and hypercoagulable states have been linked to cognitive impairment [4].

Our study has several limitations. Due to the observational nature of the study, residual confounding is a potential limitation and causality cannot be established. The majority of cohort participants are older white male U.S. veterans and the results are therefore not generalizable to other populations. This study relies on measurements of serum phosphorus level obtained during routine medical care. Serum phosphorus concentration may be affected by diurnal variation, dietary intake, fasting duration, and hormonal status [41]. Our datasets did not include information on dietary phosphorus intake, urinary phosphate excretion, timing of blood draw, or levels of FGF-23. We relied on ICD-9 codes in defining major subtypes of dementia and misclassification cannot be completely ruled out. Strengths of our study include the use of large-scale health data, the use of validated definition of dementia, and multiple sensitivity analyses to test the robustness of the association found in the primary analysis [18].

In conclusion, the results of our study indicate that higher serum phosphorus levels, even if in the normal range, are associated with increased risk of incident dementia, the association is more robust in those younger than 60 years old. Our results need to be validated in other cohorts and in the general population. Clinical investigation is needed to evaluate whether dietary interventions aimed at reducing serum phosphorus levels will ameliorate the risk of dementia. Furthermore, additional experimental studies are needed to elucidate a putative mechanism(s) underpinning the association between phosphorus and dementia.

## Supporting information

S1 Table Relationship between serum phosphorus (in quintiles) and risk of incident dementia by age group (≤60, >60 and ≤70, and >70). (DOCX)
