# Supplementary material for: Serum phosphorus levels and risk of incident dementia
Source: PLoS One. 2017 Feb 2;12(2):e0171377. doi: 10.1371/journal.pone.0171377 (PMC5289565; doi:10.1371/journal.pone.0171377)
Supplement: S1 Table — (DOCX) [file pone.0171377.s001.docx]

**S1 Table: Relationship between serum phosphorus (in quintiles) and risk of incident dementia by age group (≤60, >60 and ≤70, and >70).**

|  | Quintile 1 Phosphorus ≤ 2.9 mg/dL | Quintile 2 Phosphorus > 2.9 and ≤ 3.2 mg/dLl | Quintile 3 Phosphorus > 3.2 and ≤ 3.5 mg/dL | Quintile 4 Phosphorus > 3.5 and ≤ 3.9 mg/dL | Quintile 5 Phosphorus > 3.9 mg/dL |
| --- | --- | --- | --- | --- | --- |
|  | Hazard Ratio (95% Confidence Interval) | | | | |
| Age ≤ 60 | 1.08 (0.94, 1.25) | 1 | 1.10 (0.95, 1.27) | 1.21 (1.05, 1.40) | 1.29 (1.12, 1.48) |
| 70 ≥ Age > 60 | 1.07 (0.98, 1.18) | 1 | 0.96 (0.87, 1.06) | 1.07 (0.97, 1.18) | 1.21 (1.09, 1.34) |
| Age >70 | 1.08 (1.03, 1.14) | 1 | 1.03 (0.98, 1.09) | 1.02 (0.97, 1.07) | 1.02 (0.97, 1.08) |
| Interaction P value for 70 ≥ Age > 60 | 0.94 | NA | 0.14 | 0.16 | 0.50 |
| Interaction P value for Age > 70 | 0.97 | NA | 0.45 | 0.02 | 0.003 |

Abbreviations: HR, Hazard Ratio; CI, Confidence interval; NA, Not applicable.

Model adjusted for age group, race, gender, diabetes mellitus, cardiovascular disease, peripheral artery disease, hypertension, atrial fibrillation, depression, liver cirrhosis, baseline eGFR, number of eGFR measurements and number of hospitalizations. Additionally, interaction between age group and phosphorus quintiles were added to the model.

Phosphorus quintile 2 was used as the phosphorus reference group and age ≤ 60 was used as the age reference group.
